# Squama Manitis Extract Exhibits Broad-Spectrum Antibacterial Activity Through Energy and DNA Disruption Mechanisms

**DOI:** 10.3390/biology14080949

**Published:** 2025-07-28

**Authors:** Li Chen, Kunping Song, Mengwei Cheng, Aloysius Wong, Xuechen Tian, Yixin Yang, Mia Yang Ang, Geok Yuan Annie Tan, Siew Woh Choo

**Affiliations:** 1 Department of Biology, College of Science and Technology, Wenzhou-Kean University, 88 Daxue Road, Ouhai, Wenzhou 325060, China; s2040336@siswa.um.edu.my (L.C.); 1162627@wku.edu.cn (K.S.); 1190912@wku.edu.cn (M.C.); aloysiusw@wku.edu.cn (A.W.); tianxuechen@wku.edu.cn (X.T.); yyang@kean.edu (Y.Y.); 2 Institute of Biological Sciences, Faculty of Science, Universiti Malaya, Kuala Lumpur 50603, Malaysia; 3 Zhejiang Bioinformatics International Science and Technology Cooperation Center, 88 Daxue Road, Ouhai, Wenzhou 325060, China; 4 Zhejiang Province-Malaysia International Joint Laboratory for Modern Agriculture and Microbial Innovation, Wenzhou-Kean University, Ouhai, Wenzhou 325060, China; 5 Department of Diagnostic and Allied Health Science, Faculty of Health and Life Sciences, Management and Science University, Shah Alam 40100, Malaysia; angmiayang@msu.edu.my

**Keywords:** Squama Manitis, multi-target antimicrobial, biofilm disruption, antimicrobial resistance, traditional medicine bioprospecting

## Abstract

The global antimicrobial resistance crisis demands innovative strategies to combat bacterial infections, including those caused by drug-sensitive pathogens that evade treatment through biofilm formation or metabolic adaptations. Here, we demonstrate that Squama Manitis extract (SME)—a traditional Chinese medicine component—exhibits broad-spectrum bactericidal activity against clinically significant pathogens, including both Gram-positive (*Staphylococcus aureus*) and Gram-negative (*Escherichia coli*) species (MIC = 31.25 mg/mL), achieving significant reduction in bacterial viability within 24 h. Through integrated multi-omics analysis combining scanning electron microscopy and RNA sequencing, we reveal SME’s unprecedented tripartite mechanism of action: (1) direct membrane disruption causing cell envelope collapse, (2) metabolic paralysis through coordinated suppression of TCA cycle and fatty acid degradation pathways, and (3) inhibition of DNA repair systems (SOS response and recombination downregulation). Despite its potent activity, SME shows low cytotoxicity toward mammalian cells (>90% viability) and can penetrate Gram-negative outer membranes. These features highlight SME’s potential to address drug-resistant infections through synthetic lethality across stress response, energy metabolism, and DNA integrity pathways. While advocating for synthetic alternatives to endangered animal products, this study establishes SME as a polypharmacological template for resistance-resilient antimicrobial design, demonstrating how traditional knowledge and modern systems biology can converge to guide sustainable anti-infective development.

## Simple Summary

Antibiotic resistance is making it harder to treat bacterial infections, creating a serious threat to global health. In this study, we explored the antibacterial effects of an extract from Squama Manitis, a substance used in traditional Chinese medicine. Our goal was to understand how it kills harmful bacteria. We found that the extract works in several ways: it damages the outer layer of bacteria, stops them from making energy, and prevents them from repairing their DNA. It also showed strong effects against both common types of bacteria and was safe for human cells. These findings suggest that this natural product could inspire new types of antibiotics that are harder for bacteria to resist, while also supporting the use of traditional knowledge in modern medicine and promoting conservation by pointing toward safe synthetic alternatives.

## 1. Introduction

The global antimicrobial resistance (AMR) crisis represents one of the most urgent threats to modern medicine, with resistant pathogens causing an estimated 1.27 million deaths annually and threatening to render common infections untreatable [1]. Current antibiotic development pipelines are critically insufficient, with only a handful of novel mechanisms reaching clinical trials despite decades of investment [2]. The predominance of single-target antibiotics has inadvertently accelerated resistance evolution, as bacteria can overcome these agents through relatively simple genetic modifications affecting specific molecular targets [3,4].

Natural products have historically provided two-thirds of clinically approved antibiotics and continue to offer the most promising avenue for discovering genuinely novel antimicrobial mechanisms [5,6]. Unlike synthetic compounds that typically target single pathways, natural antimicrobials often exhibit polypharmacological effects that create formidable barriers to resistance development [4]. This multi-target approach represents a paradigm shift from conventional drug design, potentially offering solutions to the resistance crisis that single-target strategies cannot provide.

Among natural product sources, traditional Chinese medicine (TCM) represents an particularly underexploited reservoir of antimicrobial innovation, with documented therapeutic efficacy spanning millennia and sophisticated understanding of synergistic compound interactions [7]. Modern molecular investigations have begun to validate TCM mechanisms at cellular and organismal levels, revealing complex bioactive networks that often surpass single-compound efficacy [8,9,10,11]. TCM possesses a variety of biological properties that have the potential to discover or develop effective and safer drugs [12]. Among TCM components, Squama Manitis (pangolin scales) has demonstrated antimicrobial properties for over 1000 years, yet systematic mechanistic investigation remains absent despite recent pharmacological characterizations [13,14,15]. Critically, while pangolins face extinction due to overexploitation, understanding their bioactive mechanisms could enable synthetic replication without relying on endangered species—addressing both conservation concerns and therapeutic needs.

Here, we present the first comprehensive multi-omics analysis of Squama Manitis extract (SME) against the clinically significant pathogens *Escherichia coli* and *Staphylococcus aureus*, selected as WHO priority pathogens representing Gram-negative and Gram-positive challenges with extensive resistance profiles. Through integrated microbiological, ultrastructural, and transcriptomic approaches, we elucidate SME’s unique multi-target mechanism and demonstrate how traditional knowledge can guide sustainable antimicrobial development. This work establishes a conservation-conscious framework for bioinspired drug discovery that could transform our approach to combating antimicrobial resistance.

## 2. Materials and Methods

### 2.1. Sample Preparation

All Squama Manitis (SM) samples were purchased before June 2020 from Tong Ren Tang pharmaceutical company (Beijing, China). These samples were obtained from legally marketed traditional medicine products purchased through commercial channels in China. No wild pangolins were collected, handled, or harmed for the purpose of this research, and no additional trade in pangolin-derived materials was conducted as part of the study. This work does not promote the medicinal use of pangolin scales. Instead, it aims to characterize their bioactive components and antibacterial mechanisms to support the development of sustainable alternatives that may help reduce demand for wild pangolin products and contribute to species conservation. Sample preprocessing followed established protocols with modifications [16]. Briefly, SM samples were surface-sterilized by vigorous rinsing and wiping with 75% ethanol, followed by sterile water washing to remove surface contaminants. Samples were dried in a 60 °C incubator overnight, then pulverized using a sterile masher (Little Bear Electric Co., Foshan, China) that was pre-sterilized with 75% ethanol. The resulting powder was collected under sterile conditions and stored at −20 °C until use in subsequent experiments.

### 2.2. Preparation of SME

SME was prepared using aqueous extraction methods. Four grams of ground SM powder were suspended in 20 mL of sterile distilled water and incubated in a water bath at 80 °C for 48 h with periodic agitation. The resulting extract was cooled to room temperature and filtered through a 0.22 μm sterile microporous membrane (Millipore, Billerica, MA, USA) to remove particulate matter and achieve sterility. The final filtrate yielded a stock SME solution with a nominal concentration of 200 mg/mL (assuming complete extraction). Working concentrations were prepared by serial two-fold dilutions in sterile water, and all solutions were stored at 4 °C and used within one week of preparation to maintain stability.

### 2.3. Cell Culture

*E. coli* ATCC 25922 and *S. aureus* ATCC 25923 were employed in this study. Bacteria were routinely cultured in lysogeny broth (LB, Qingdao Hope Bio-Technology Co., Ltd., Qingdao, China) or on solidified lysogeny agar (LB containing 15 g L^−1^ Bacto-agar) (Qingdao Hope Bio-Technology Co., Ltd., Qingdao, China). For MIC and MBC assays, individual colonies from agar plates were picked, transferred to 2 mL LB, and incubated at 37 °C with shaking at 200 rpm for 12 h. Cultures were diluted to a concentration of 0.5 McFarland turbidity standard, containing approximately 1 × 10^8^–2 × 10^8^ CFU/mL.

### 2.4. Antibacterial Assays

Broth microdilution assays were used to investigate the MIC, MBC, and bacterial growth curves.

### 2.5. MIC and MBC Determination

In a 96-well plate, a total volume of 200 μL of LB broth medium and sterile water were added as a negative control group, and the same volume of prepared bacterial suspension (final concentration of 1 × 10^5^ CFU/mL) was used as a positive control. The same volume of prepared bacterial suspension and SME were added as treatment groups so that the final concentrations of SME were 125.00 mg/mL, 62.50 mg/mL, 31.25 mg/mL, 15.63 mg/mL, 7.81 mg/mL, 3.91 mg/mL, 1.95 mg/mL, and 0.98 mg/mL, respectively. The plates were incubated with shaking at 200 rpm in a 37 °C incubator for 24 h. The bacterial growth was monitored by measuring the optical density at 600 nm (OD600) using a Varioskan Flash microplate reader (Thermo Scientific, Shanghai, China). The MIC value was determined to be the lowest concentration at which no growth of the organism was detected. Following the measurement of the SME’s MIC, 50 µL aliquots of all tubes with no obvious bacterial growth were inoculated onto LB agar plates (Qingdao Hope Bio-Technology Co., Ltd., Qingdao, China) and incubated for 24 h at 37 °C. The MBC endpoint was defined as the presence of fewer than 5 colonies on an agar plate at the lowest concentration of an antimicrobial agent. All experiments were conducted in three biological replicates.

### 2.6. Growth Curve Analysis

Bacterial suspensions (1 × 10^5^ CFU/mL) in 96-well plates were treated with SME at MIC or sterile water (control). Growth was monitored by OD600 measurements at 3, 6, 9, 12, and 24 h using a Varioskan Flash microplate reader (Thermo Scientific, Shanghai, China). All experiments included three biological replicates.

### 2.7. Antibacterial Activity Against Additional Bacterial Strains

Broth microdilution assays assessed SME’s activity against additional bacterial species, broth microdilution assays were performed on Gram-negative (*A. hydrophila*, *A. lwoffii*, *P. aeruginosa*) and Gram-positive (*B. cereus*) strains. Bacterial suspensions (1 × 10^5^ CFU/mL) were prepared in 96-well plates with a total volume of 200 μL per well. The suspensions were treated with SME (20 mg/mL or 40 mg/mL), or sterile water (control). The plates were incubated with shaking at 200 rpm at 37 °C for 24 h. Bacterial growth was monitored by measuring the OD600 using a Varioskan Flash microplate reader (Thermo Scientific, Shanghai, China), and the growth rate (%) was calculated relative to control. All experiments were conducted in three biological replicates.

### 2.8. Cytotoxicity Assay

Human HaCaT cell viability was assessed via CCK-8 assay. Cells (1 × 10^4^/well) were seeded in 96-well plates overnight, then treated with SME (3.91–125.00 mg/mL) for 24 h. After adding CCK-8 solution (Dojindo Laboratories, Kumamoto, Japan), plates were incubated for 2 h at 37 °C before measuring absorbance at 450 nm (Thermo Scientific, Shanghai, China). Viability was normalized to untreated controls. All experiments were conducted in three biological replicates.

### 2.9. Scanning Electron Microscopy (SEM)

Logarithmic growth stage *E. coli* and *S. aureus* cultures (diluted to ~10^5^ CFU/mL) were untreated (control) and treated with SME at its MIC value and incubated at 37 °C for 24 h. After removal of the medium, these bacterial cells were collected, washed twice with PBS (0.1M), and fixed with 2.5% glutaraldehyde. The stationary liquid was discarded, and the samples were rinsed three times for 15min with phosphoric acid–sulfuric acid buffer solution (0.1 M, pH = 7.0). After the samples were fixed in 1% osmic acid solution for 1–2 h, we rinsed the samples three times for 15 min with phosphate buffer (0.1 M, pH = 7.0). Samples were dehydrated in a gradient of 30%, 60%, 70%, 80%, 90%, 95%, and 100% ethanol for 15 min at each concentration. The samples were treated with a mixture of ethanol and isoamyl acetate (V/V = 1/1) for 30 min and pure isoamyl acetate for 1 h or overnight. The sample was dropped on the silicon substrate and dried at the critical point by K850 Critical Point Dryer (Quorum Technologies Ltd., Lewes, UK). SEM micrographs were taken at 10,000×, 30,000×, and 60,000× magnifications using a Field-Emission Scanning Electron Microscope (SU8010, Hitachi, Ltd., Tokyo, Japan). All experiments were performed using four biological replicates per group.

### 2.10. RNA Isolation and Integrity Analysis

Log-phase *E. coli* were treated with SME (31.25 mg/mL) for 24 h at 37 °C, and the same volume of sterile water was used as the control. After that, the total RNA of each sample was extracted from cells using TRIzol reagent (Ambion Inc., Austin, TX, USA). The total RNA of each sample was quantified and qualified by an Agilent 2100/2200 Bioanalyzer (Agilent Technologies, Palo Alto, CA, USA), NanoDrop (Thermo Fisher Scientific Inc., Waltham, MA, USA), and 1% agarose gel.

### 2.11. Library Construction and Whole-Transcriptome Sequencing

A total of 1 μg total RNA was used for the following library preparation. Next-generation sequencing library preparations were constructed according to the manufacturer’s protocol. The rRNA was depleted from total RNA using the QIAseq FastSelect −5S/16S/23S Kit (Qiagen, Valencia, SC, USA). The depleted ribosomal RNA was then fragmented and reverse-transcribed. First-strand cDNA was synthesized using ProtoScript II Reverse Transcriptase (New England BioLabs Inc., Ipswich, MA, USA) with random primers and Actinomycin D. The second-strand cDNA was synthesized using Second Strand Synthesis Enzyme Mix (include dACGTP/dUTP). The purified double-stranded cDNA by magnetic beads in the QIAseq FastSelect −5S/16S/23S kit (Qiagen, Valencia, SC, USA) was then treated with End Prep Enzyme Mix to repair both ends and add a dA-tailing in one reaction, followed by a T-A ligation to add adaptors to both ends. Size selection of Adaptor-ligated DNA was then performed using beads, and fragments of ~400 bp (with the approximate insert size of 300 bp) were recovered. The dUTP-marked second strand was digested with Uracil-Specific Excision Reagent enzyme. Each sample was then amplified by PCR using P5 and P7 primers (P5 primer: 5′-AATGATACGGCGACCACCGA-3′; P7 primer: 5′-CAAGCAGAAGACGGCATACGA-3′), with both primers carrying sequences which can anneal with flow cell to perform bridge PCR and P5/P7 primer carrying index allowing for multiplexing. The VAHTS^®^ Universal V8 RNA-seq Library Prep Kit (Vazyme Biotech Co., Ltd., Nanjing, China) was used to perform PCR amplification. The PCR products were cleaned up using beads, validated using an Qsep100 (Bioptic, Taiwan, China), and quantified by Qubit3.0 Fluorometer (Invitrogen, Carlsbad, CA, USA). The libraries were loaded on an Illumina Novaseq 6000 instrument (Illumina, San Diego, CA, USA) and sequenced using a 2 × 150 bp paired-end (PE) strategy according to manufacturer’s instructions. Image analysis and base calling were performed by the Zebeacall on the MGI2000 instrument (BGI, Shenzhen, China).

### 2.12. Quality Control and Mapping

To ensure the high quality of our sequencing data, all raw reads were processed by Cutadapt [17] (version 1.9.1, phred cutoff: 20, error rate: 0.1, adapter overlap: 1bp, min. length: 75, the proportion of N: 0.1). Poor-quality bases or reads lower than 20 and adapter sequences were removed. All processed reads were mapped to reference genome sequence (*E. coli* ATCC 25922; accession number = ASM74325v1) which was downloaded from NCBI. Bowtie2 (v2.2.6) was used to index reference genome sequences before aligning reads to the reference genome (accession number = ASM74325v1) [18].

### 2.13. Differential Gene Expression Analysis

HTSeq (v0.6.1p1) [19] was used to estimate gene expression levels from the pair-end clean reads. To identify differentially expressed genes, we compared the gene profiles between controls and treatment groups using the well-established DESeq2 Bioconductor package (v1.16.1). A gene was defined as significantly differentially expressed if its fold change ≥2 and False Discovery Rate (FDR) < 0.05.

### 2.14. Functional Enrichment Analysis

Gene Ontology (GO) enrichment was performed using the GOSeq (v1.34.1) [20]. GO terms with a significant *p*-value less than 0.05 were selected for analysis. TopGO was used to generate Directed Acyclic Graphs (DAGs) to visualize the hierarchical structure of enriched GO terms. KOBAS (2.0) [21] was used for pathway significant enrichment analysis, which took pathways in the Kyoto Encyclopedia of Genes and Genomes (KEGG) database [22] as units, and applied the hypergeometric test to find the pathways that showed significant enrichment in differentially expressed genes compared with the whole-genome background.

### 2.15. PPI Network Analysis

To study the protein–protein interaction (PPI) networks of differentially expressed genes, we performed a network analysis by using the STRING (Search Tool for the Retrieval of Interacting Genes/Proteins) database version 11.0 with default settings [23,24]. The network was clustered using the MCL clustering method provided under the STRING Clusters tab with an inflation parameter of 3 [25]. The enrichment analysis (biological process) was performed and results with multiple testing corrections were used for further analysis. FDR threshold <0.05 was used to define significant processes, which were color-coded using the STRING analysis tool tab. To improve visual clarity, only the largest cluster from the upregulated and downregulated DEG networks was displayed, and functional clusters were color-coded.

### 2.16. Statistical Analysis

Antibacterial rates and the growth curves of *E. coli* and *S. aureus* were expressed as mean ± SD based on three independent biological replicates. For the antibacterial rates and cytotoxicity assay, differences between treatment groups and the control were assessed using one-way ANOVA followed by Dunnett’s post hoc test. For the growth curve, differences across doses and time points were analyzed using two-way repeated measures ANOVA followed by Tukey’s post hoc test. Statistical significance was set at *p* < 0.05, with significance levels denoted as * *p* < 0.05, ** *p* < 0.01, *** *p* < 0.001, and **** *p* < 0.0001 in figures and tables. All statistical analyses were performed using GraphPad Prism 9 (GraphPad, San Diego, CA, USA).

## 3. Results

### 3.1. SME Exhibits Potent, Selective Antibacterial Activity

**Figure 1:**
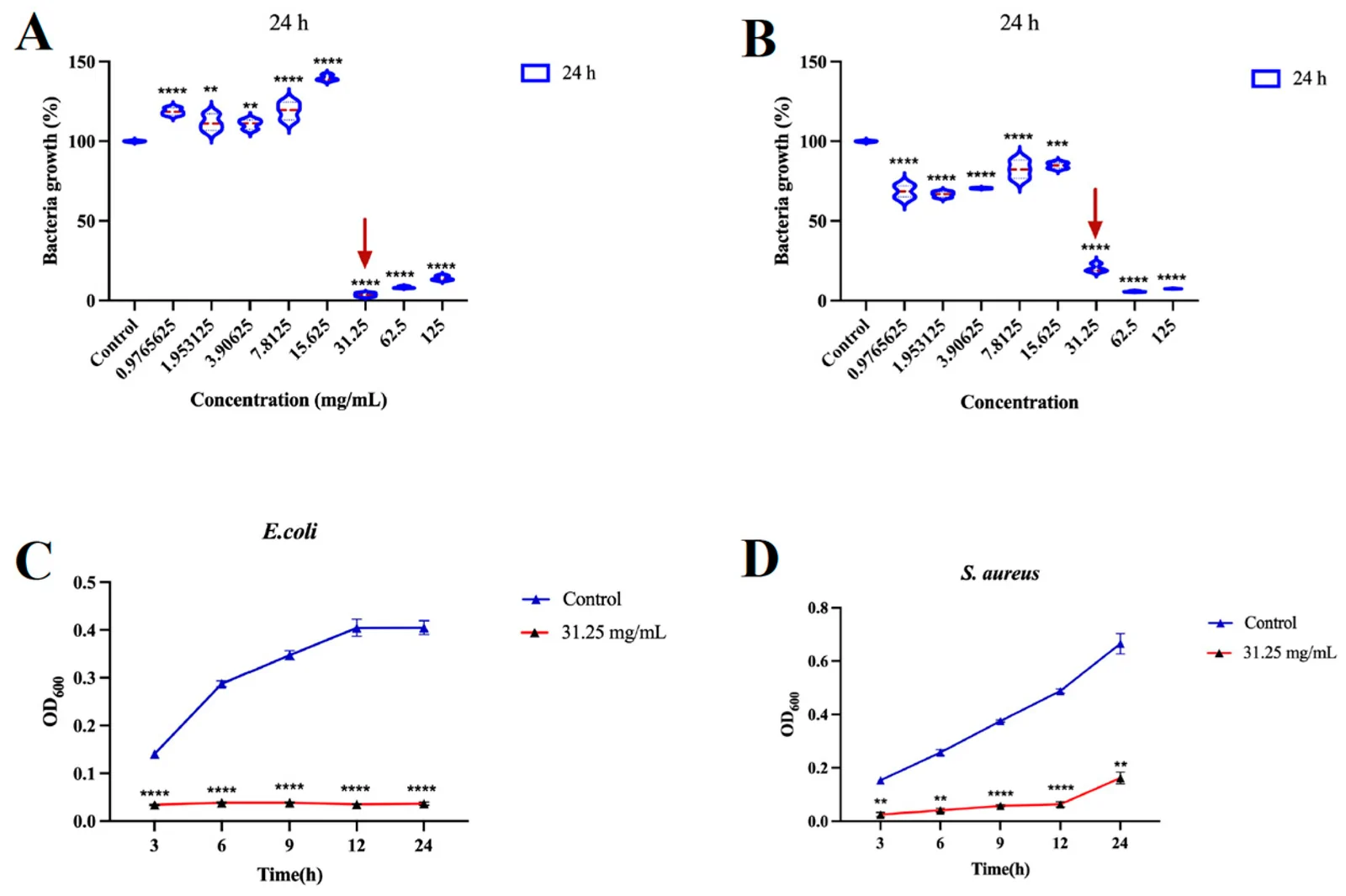
**SME exhibits potent antibacterial activity against *E. coli* and *S. aureus*.** Minimum inhibitory concentrations (MICs) of SME against *E. coli* (**A**) and *S. aureus* (**B**) determined by broth microdilution assay after 24 h treatment. Time-kill kinetics of *E. coli* (**C**) and *S. aureus* (**D**) treated with SME at the MIC. Data represent mean ± s.d. of three biological replicates. ** *p* < 0.01, *** *p* < 0.001, and **** *p* < 0.0001.

Our systematic evaluation of Squama Manitis extract (SME) revealed robust antibacterial properties against diverse bacterial pathogens. Using standardized broth microdilution assays, we determined SME’s minimum inhibitory concentration (MIC) to be 31.25 mg/mL against both *Escherichia coli* (ATCC 25922) and *Staphylococcus aureus* (ATCC 25923) after 24 h of incubation (Figure 1A,B). This consistent activity across Gram-positive and Gram-negative species is particularly significant given the intrinsic resistance of Gram-negative species conferred by their outer membrane barrier.

Time-kill kinetic studies demonstrated rapid bactericidal action, achieving significant reduction in viable counts within 24 h, supporting its potential use in treating acute infections that require rapid pathogen clearance (Figure 1C,D). Minimum bactericidal concentration (MBC) analysis revealed low MBC/MIC ratios of 1.0 for *E. coli* and 2.0 for *S. aureus* (Table 1), with the former indicating SME’s ability to overcome the protective outer membrane of Gram-negative bacteria—a major challenge for many conventional antibiotics [26].

To assess broad-spectrum potential, SME was tested against additional clinically relevant strains (Figure S1). Among Gram-negative bacteria, *Aeromonas hydrophila* (*A. hydrophila*) showed 91.25% and 94.57% growth inhibition at 20 mg/mL and 40 mg/mL, respectively; *Acinetobacter lwoffii* (*A. lwoffii*) displayed 88.45% and 97.27% inhibition at corresponding concentrations; *Pseudomonas aeruginosa* (*P. aeruginosa*) exhibited moderate susceptibility with 43.51% and 91.43% inhibition. For Gram-positive *Bacillus cereus* (*B. cereus*), 86.32% growth reduction was observed at 20 mg/mL. Notably, *A. lwoffii* demonstrated near-complete growth arrest (97.27%) at 40 mg/mL, suggesting species-specific vulnerabilities to SME’s multi-target mechanism.

Critically for therapeutic development, SME exhibited excellent selectivity with minimal cytotoxicity, retaining 101% viability in human HaCaT keratinocytes at the bactericidal MIC of 31.25 mg/mL and approximately 93% at 125.00 mg/mL across the tested range of 3.91–125.00 mg/mL, relative to the untreated control (all no significant different) (Figure S2). This favorable therapeutic index, indicating selective toxicity toward bacterial over mammalian cells, underscores SME’s potential as a safe and effective antimicrobial candidate warranting further clinical investigation [27].

### 3.2. SME Disrupts Bacterial Cell Envelope Integrity

**Figure 2:**
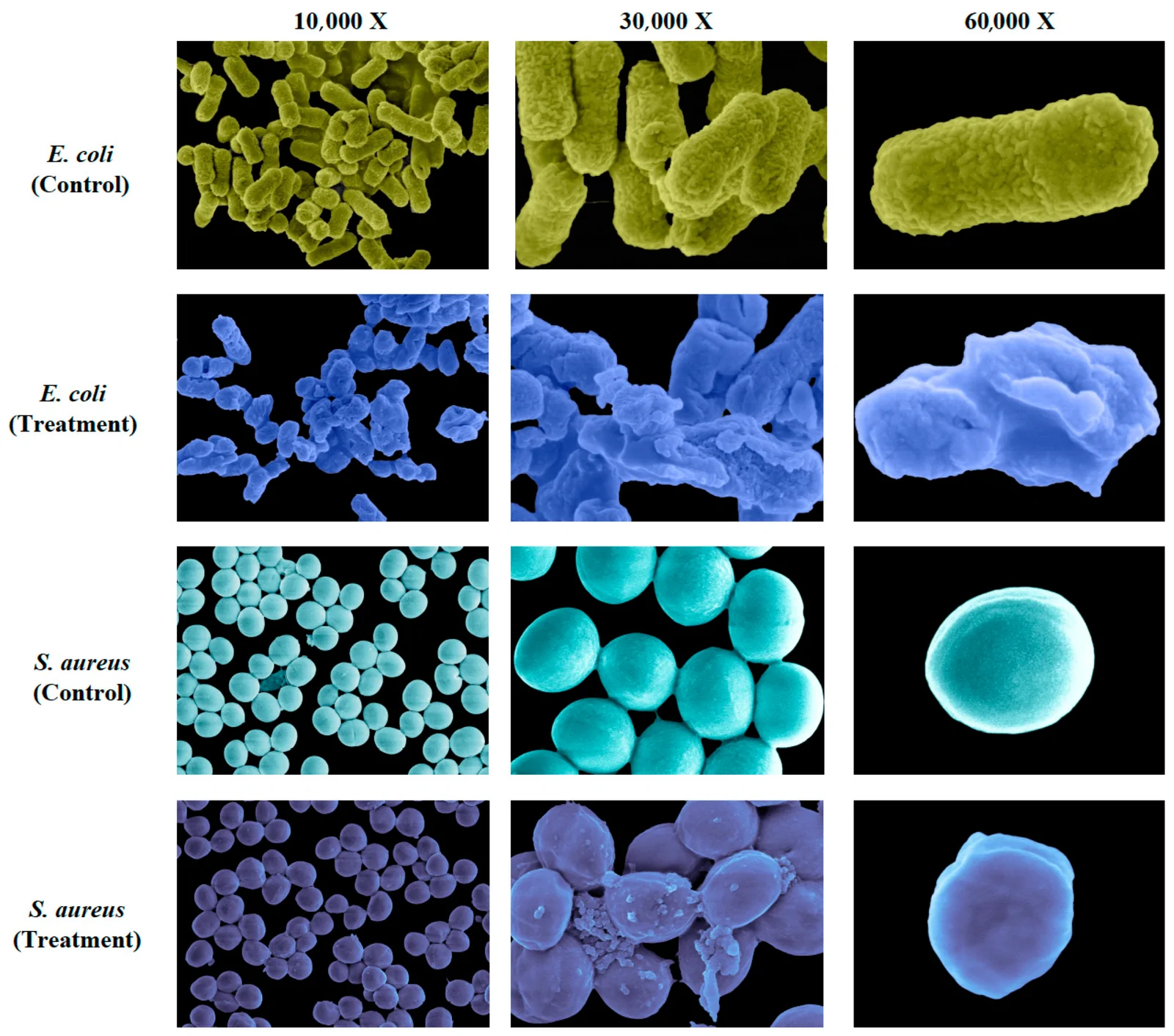
**SME disrupts *E. coli* cell envelope integrity**. Representative scanning electron micrographs of untreated and SME-treated *E. coli* and *S. aureus* cells after 24 h. SME was used at the MIC of 31.25 mg/mL. Magnifications: 10,000× (first column), 30,000× (second column), 60,000× (third column).

To gain further insight into SME’s antibacterial mode of action, we examined its impact on bacterial cell morphology and ultrastructure using scanning electron microscopy (SEM). Untreated *E. coli* cells maintained their characteristic rod-shaped morphology with smooth, intact surfaces, while *S. aureus* exhibited typical spherical forms with preserved cell wall integrity (Figure 2). In stark contrast, SME-treated cells showed extensive ultrastructural damage, including severe membrane crumpling, deep surface invaginations, and loss of cytoplasmic content, indicating extensive membrane disruption. Gram-positive *S. aureus* displayed similarly dramatic damage patterns, with surface roughening, multiple rupture sites, and cytoplasmic leakage.

The universal nature of this damage across bacterial classifications, despite their structural differences in cell wall composition, suggests SME’s mechanism involves fundamental disruption of membrane integrity. This membrane-targeting activity differs fundamentally from conventional antibiotics that inhibit specific enzymes or cellular processes [28], potentially circumventing existing resistance mechanisms that rely on single-target modifications. The observed damage patterns support a model where SME may either intercalate into membrane phospholipids or inhibit cell wall biosynthesis pathways, leading to catastrophic loss of membrane integrity and rapid cell death.

### 3.3. Transcriptomic Repogramming Reveals SME’s Multi-Faceted Antibacterial Mechanism

**Figure 3:**
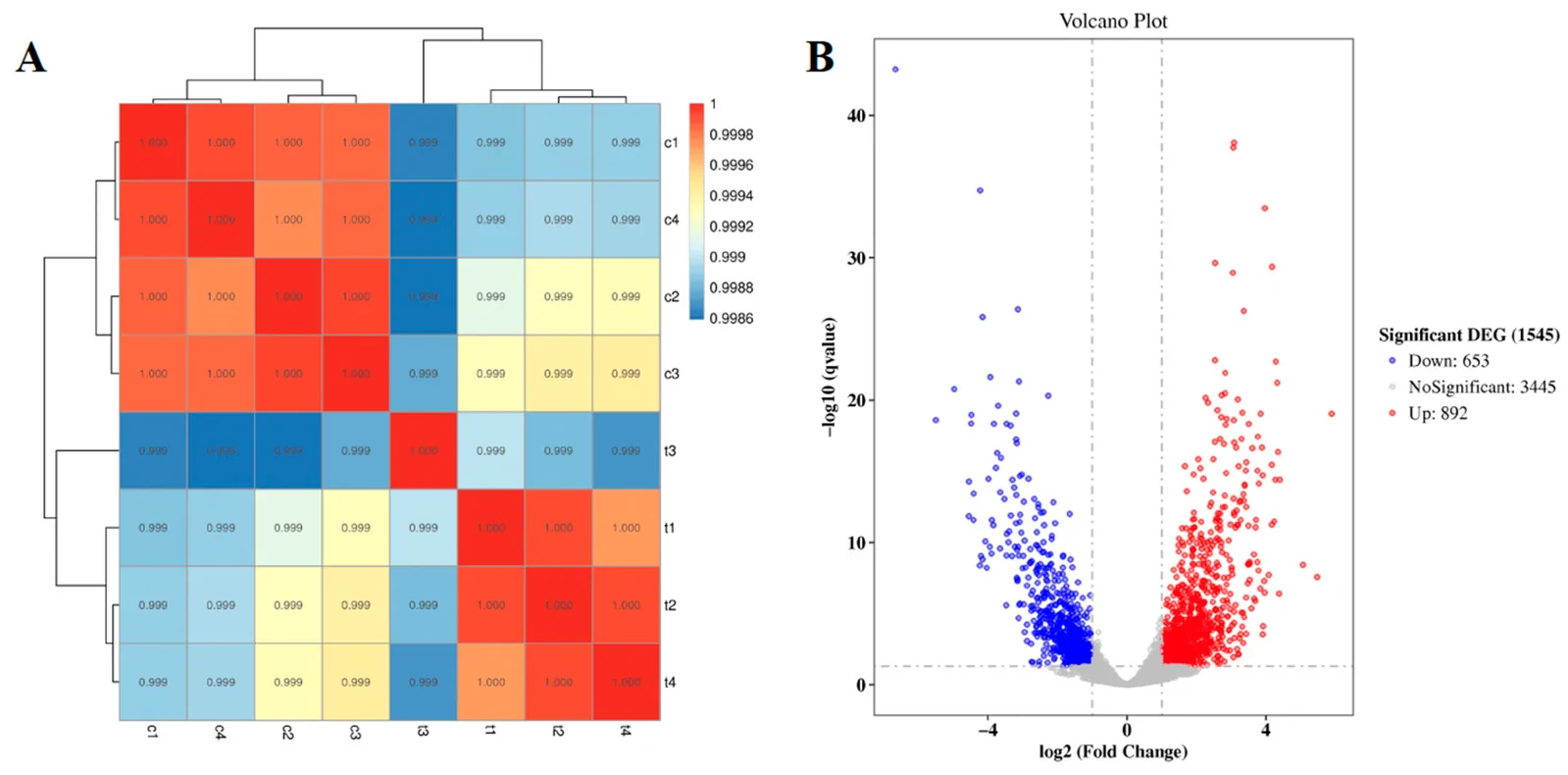
**Transcriptional landscape of SME-treated *E. coli*.** (**A**) Principal component analysis demonstrating clear separation between treatment groups. (**B**) Volcano plot of differentially expressed genes (DEGs) showing log_2_fold changes versus statistical significance (−log_10_q-value). Red: upregulated DEGs; blue: downregulated DEGs; gray: non-significant genes.

To comprehensively characterize SME’s mode of action, we performed genome-wide transcriptional profiling of *E. coli* exposed to SME at its MIC (31.25 mg/mL) for 24 h. RNA sequencing generated deep transcriptional profiles with approximately 15 million high-quality reads per sample, and showed exceptional alignment rates (>97%) to the *E. coli* reference genome (Tables S1–S3). Unsupervised principal component analysis revealed clear separation between treated and control groups (Figure 3A), demonstrating SME’s profound impact on global gene expression patterns. Differential expression analysis identified 1545 significantly altered genes (FDR < 0.05), with 892 genes upregulated and 653 downregulated, indicating widespread reprogramming of bacterial physiology in response to SME exposure (Figure 3B, Tables S4 and S5).

**Figure 4:**
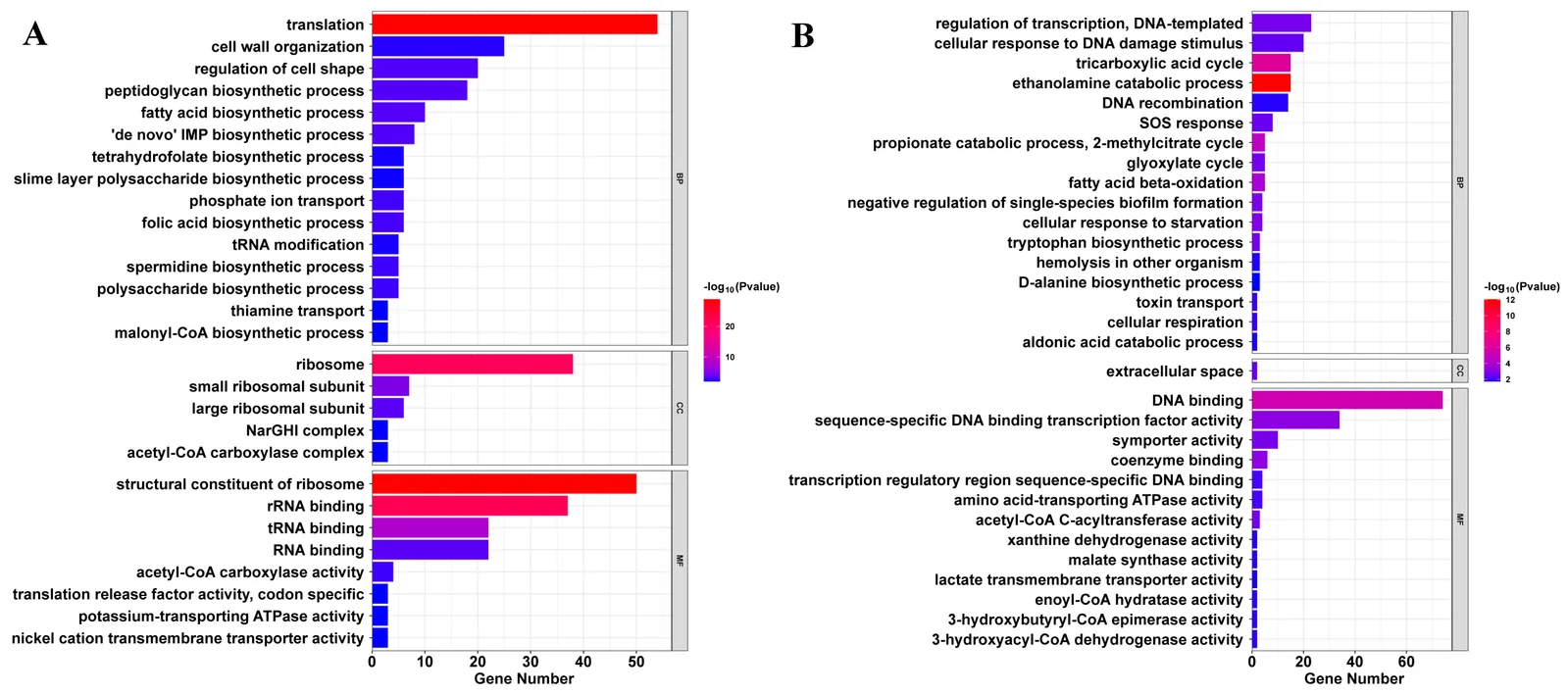
**Functional enrichment analysis of differentially expressed genes in SME-treated *E. coli***. (**A**) Upregulated genes are mainly enriched in translation and cell envelope biosynthesis, including ribosome and peptidoglycan pathways. (**B**) Downregulated genes are enriched in central metabolism and DNA repair, including the TCA cycle, ethanolamine utilization, and SOS response. Bubble size corresponds to the number of associated DEGs; color intensity reflects statistical significance (−log_10_*p*-value).

Gene Ontology (GO) enrichment analysis demonstrated that the upregulated gene set was significantly enriched in biological processes related to translation, including translation (GO:0006412), ribosome (GO:0005840), and structural constituents of ribosome (GO:0003735), indicating enhanced protein synthesis activity (Figure 4A and Table S6). In parallel, multiple pathways associated with cell envelope biosynthesis and maintenance were also upregulated, such as cell wall organization (GO:0071555), peptidoglycan biosynthetic process (GO:0009252), and regulation of cell shape (GO:0008360). The co-induction of fatty acid and tetrahydrofolate biosynthetic processes (GO:0006633 and GO:0046654) suggests activation of membrane lipid and cofactor synthesis pathways, likely as a compensatory response to SME-induced cell envelope disruption. These transcriptional adaptations, together with SEM observations of extensive cell envelope damage, imply that SME triggers cell wall perturbation, prompting *E. coli* to enhance envelope reconstruction and translational output as a stress adaptation strategy, consistent with well-characterized compensatory responses to membrane damage and protein denaturation [29].

Conversely, downregulated genes clustered in three functionally interconnected categories critical for bacterial survival (Figure 4B and Table S7). Central carbon metabolism, including the tricarboxylic acid (TCA) cycle (GO:0006099), glycolysis cycle (GO:0006097), fatty acid beta-oxidation (GO:0006635), ethanolamine catabolic process (GO:0046336), 2-methylcitrate cycle (GO:0019629), and cellular respiration (GO:0045333), was significantly repressed, effectively starving cells of energy and biosynthetic precursors. Biosynthetic pathways for amino acids and nucleotides, along with related enzymes such as xanthine dehydrogenase activity (GO:0004854), were similarly suppressed, while DNA replication/repair systems—particularly the SOS response (GO:0009432), DNA recombination (GO:0006310), and DNA binding (GO:0003677) including sequence-specific DNA binding transcription factor activity (GO:0003700)—exhibited marked reductions, indicating impaired genome integrity maintenance. This pattern of transcriptional repression mirrors aspects of the bacterial stringent response, but with several critical distinctions, including deeper metabolic suppression and the unique combination with cell envelope stress signals, as evidenced by the upregulation of peptidoglycan biosynthetic process (GO:0009252) and fatty acid biosynthetic process (GO:0006633) [30]. The coordinated nature of these effects creates a synthetic lethal scenario, where concurrent disruption of normally redundant systems proves fatal to bacterial cells [31].

### 3.4. KEGG Pathway Analysis Reveals SME’s Coordinated Multi-Target Mechanism

**Figure 5:**
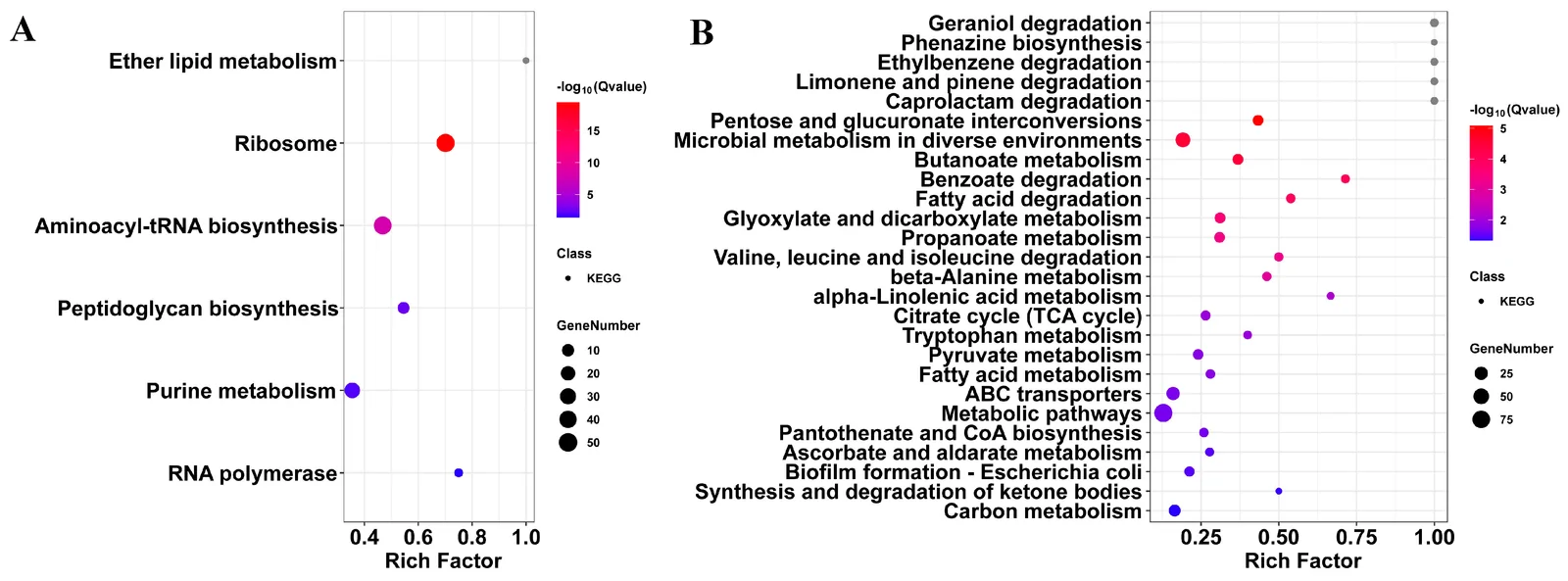
**KEGG pathway analysis of SME-responsive genes in *E. coli*.** (**A**) Upregulated pathways include ribosome, tRNA biosynthesis, and cell wall assembly. (**B**) Downregulated pathways include the TCA cycle, fatty acid degradation, and biofilm formation. Circle size represents the rich factor (DEGs per pathway); color gradient indicates enrichment significance (−log_10_Q-value).

To systematically characterize SME’s antibacterial mechanism, KEGG pathway enrichment analysis was performed on both upregulated and downregulated gene sets, with results presented in Figure 5A,B and detailed in Tables S8 and S9. Upregulated pathways revealed the enhanced activation of protein synthesis machinery, including significant enrichment of ribosome (ko03010) and aminoacyl-tRNA biosynthesis (ko00970), suggesting SME-exposed cells may induce compensatory upregulation of translational pathways in response to cellular stress [32,33]. Concurrent upregulation of purine metabolism (ko00230) likely reflects increased demand for nucleotide biosynthesis to support stress adaptation. Additional enrichment of peptidoglycan biosynthesis (ko00550), RNA polymerase (ko03020), and ether lipid metabolism (ko00565) pathways indicated broader activation of cell wall assembly, transcription, and membrane remodeling processes.

In contrast, pathways in which downregulated genes were involved in TCA cycle (ko00020), carbon metabolism (ko01200), and fatty acid degradation (ko00071) were strongly inhibited, leading to energy starvation (Figure 5B and Table S9). This indicated a broad collapse of central carbon metabolism following SME treatment. The suppression of amino acid metabolism pathways (e.g., valine/leucine/isoleucine degradation (ko00280), tryptophan metabolism (ko00380), and beta-alanine metabolism (ko00410)) further supports a starvation-like state. Additionally, pathways involved in pyruvate metabolism (ko00620), propanoate metabolism (ko00640), glyoxylate and dicarboxylate metabolism (ko00630), and butanoate metabolism (ko00650) were significantly repressed, indicating further impairment of interconnected carbon fluxes and energy generation [34]. Downregulation of pantothenate and CoA biosynthesis (ko00770) and ascorbate and aldarate metabolism (ko00053) suggests compromised cofactor synthesis and redox balance. Critical to clinical relevance, biofilm formation (*E. coli*, ko02026) pathways showed pronounced suppression, disrupting bacterial communication networks that typically confer antibiotic tolerance [35].

This coordinated pathway suppression reveals SME’s sophisticated polypharmacological strategy: simultaneous membrane disruption, metabolic paralysis, cofactor depletion, and biofilm inhibition. Unlike conventional single-target antibiotics, this multi-layered approach creates synthetic lethality requiring coordinated resistance mutations across unrelated systems—a statistically improbable evolutionary scenario that positions SME as a compelling template for resistance-resilient antimicrobial design.

### 3.5. Protein–Protein Interaction (PPI) Networks Reveal SME’s Core Targets

**Figure 6:**
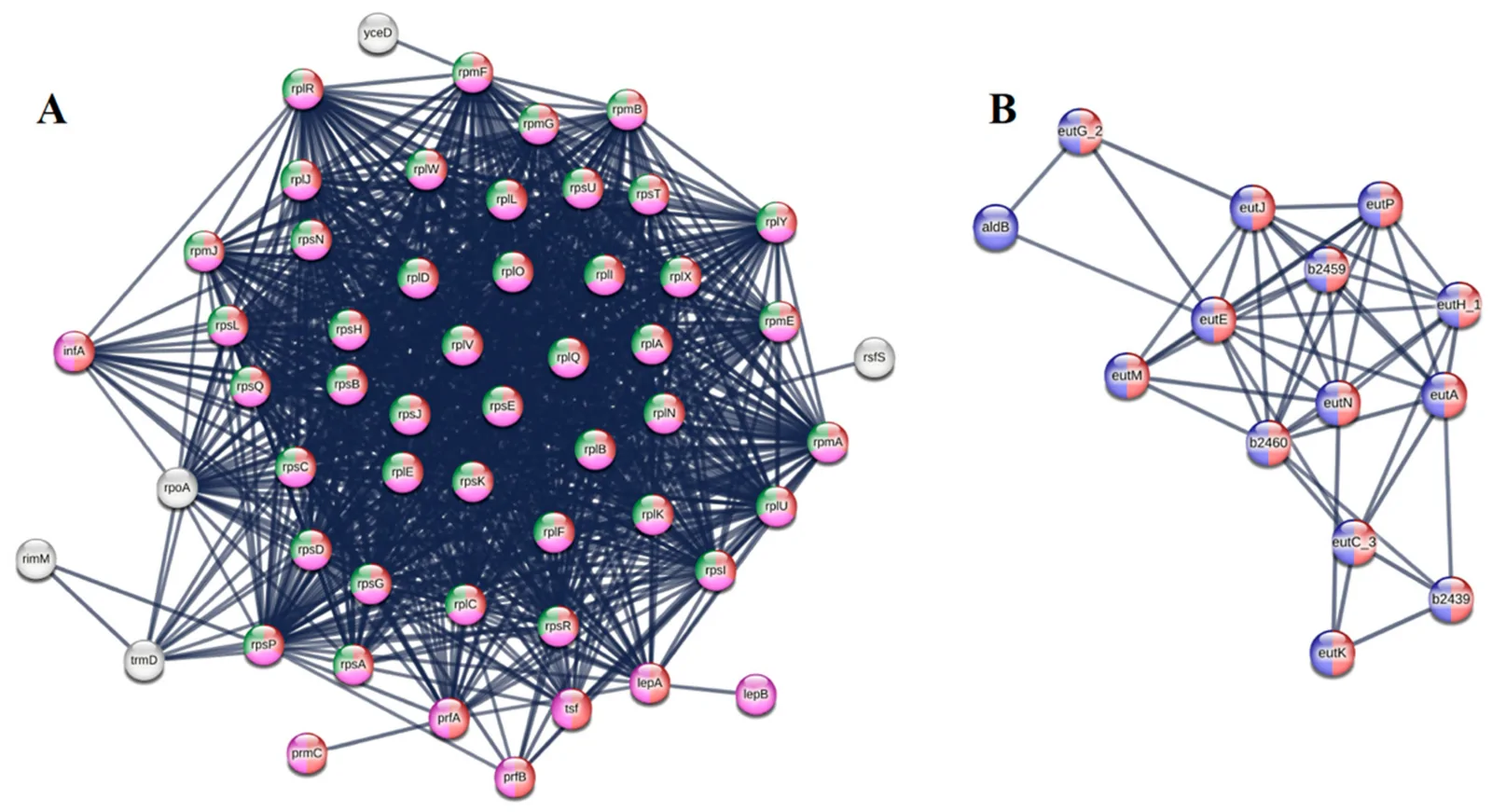
**Protein–protein interaction networks of SME-responsive genes in *E. coli*.** (**A**) Upregulated network cluster showing coordinated induction of translation machinery (red nodes), peptide metabolism (pink), and ribosome biogenesis (green). (**B**) Downregulated network cluster demonstrating suppression of ethanolamine utilization (red) and central metabolic processes (blue). Nodes represent proteins (size reflects connectivity); edges indicate functional associations (line thickness shows confidence score). All shown interactions are statistically significant (FDR < 0.05).

Protein–protein interaction network analysis identified functionally coordinated gene clusters responsive to SME treatment (Figure 6). The most prominent feature of the upregulated network was a densely interconnected cluster of 38 nodes centered around ribosomal proteins (*rplA*, *rpsA*, and *infA*). This network was significantly enriched in translation initiation and peptide metabolic processes, suggesting SME induces ribosomal stress—a response typically triggered by translation-targeting antibiotics [36].

The downregulated network revealed an equally important but contrasting pattern, with the ethanolamine utilization (eut) operon (*eutA*, *eutE*) emerging as the most connected components. As ethanolamine serves as a critical source of both carbon and nitrogen for essential biosynthetic pathways, its suppression creates a metabolic bottleneck that starves cells of energy and building blocks [37]. This dual assault on bacterial physiology—simultaneously forcing cells to increase protein production while limiting their capacity to fuel this process—creates an unsustainable biological scenario that likely contributes to SME’s rapid bactericidal effects.

These network signatures help explain SME’s effectiveness against both Gram-positive and Gram-negative pathogens despite their differing membrane architectures. The ribosomal stress response appears to be a universal bacterial reaction to SME exposure, while the metabolic disruption targets fundamental pathways conserved across species. The high connectivity of the affected network hubs suggests SME impacts central regulators of bacterial physiology rather than peripheral targets, which may account for its broad-spectrum activity.

## 4. Discussion

Our integrated multi-omics analysis reveals that SME achieves broad-spectrum antibacterial effects through a coordinated three-pronged mechanism. First, scanning electron microscopy demonstrates direct disruption of cell envelope integrity across both Gram-positive and Gram-negative species. Second, transcriptomic profiling shows simultaneous suppression of central metabolic pathways, including the TCA cycle fatty acid degradation, and pyruvate metabolism. We observed consistent suppression of key TCA cycle genes (*gltA*, *acnB*, *sucA*, *sdhA*, *sdhB*, *sdhC*, *sdhD*, and *fumC*), which are essential for energy production. Their inhibition causes severe metabolic dysfunction, termed “metabolic paralysis,” characterized by disrupted ATP generation and reduced bacterial [38,39]. The upregulation of ribosomal genes (*der*, *cgtA*, *rplJ*, *yceD*) represents a compensatory stress response to maintain basic cellular functions despite metabolic suppression [40]. Third, GO enrichment analysis revealed significant downregulation of DNA recombination, cellular response to DNA damage stimulus, and SOS response pathways, suggesting SME impairs bacterial genomic stability and stress-induced DNA repair mechanisms. This multi-target attack may induce synthetic lethality by concurrently disrupting membrane integrity, energy metabolism, and DNA integrity maintenance systems, resulting in rapid bactericidal activity. While direct functional assays (ATP measurement, DNA damage tests) were not conducted due to sample limitations, our findings align with those established in [41]. Additionally, SME treatment downregulated genes in biofilm formation pathways (KEGG analysis), providing a foundation for future validation [42]. SME’s efficacy in bacterial clearance highlights its potential as a promising framework for resistance-resilient antimicrobial development.

The clinical potential of this mechanism is enhanced by several critical findings. First, SME maintains efficacy against both Gram-positive and Gram-negative pathogens despite their differing membrane architectures, as evidenced by its remarkably low MBC/MIC ratio for *E. coli*. The MIC value of SME (31.25 mg/mL) is expected for crude extracts containing mixed bioactive and inactive constituents. The concentration reflects total extract mass, not individual compound potency. The MBC/MIC ratio of 1.0 for *E. coli* demonstrates bactericidal activity at the MIC, indicating direct killing effects despite higher concentrations required. This is consistent with other natural product screenings. This broad-spectrum activity is particularly valuable given the increasing prevalence of multi-drug-resistant infections that require empirical treatment before pathogen identification. Second, the suppression of the biofilm formation pathway suggests SME may overcome a major clinical challenge, biofilm-mediated antibiotic tolerance, that renders chronic infections particularly difficult to treat [42]. Notably, GO enrichment also revealed the inhibition of negative regulation of single-species biofilm formation, which may reflect deregulation of signaling circuits controlling biofilm homeostasis. These attributes position SME as a promising template for novel antimicrobial development, especially given the growing need for agents that can circumvent conventional resistance mechanisms.

Our parallel work identifying specific bioactive metabolites provides a path forward for sustainable therapies, where screening 33 metabolites in SM led to seven antibacterial compounds (e.g., malic acid, hippuric acid) and the subsequent development of synthetic MAC4, which demonstrates potent antibacterial activity and biofilm disruption confirmed by crystal violet staining and SEM experiments [43]. This approach represents a paradigm shift in natural product drug discovery from the direct exploitation of endangered species to understanding and replicating bioactive mechanisms through synthetic chemistry, demonstrating that traditional medicine can guide modern antimicrobial development while offering a conservation-conscious strategy to combat antibiotic resistance.

When compared to conventional antibiotics that typically target single cellular processes (β-lactams inhibit cell wall synthesis, fluoroquinolones target DNA gyrase, aminoglycosides disrupt protein synthesis), SME’s polypharmacological approach may simultaneously attack membrane integrity, central metabolism, DNA repair and transcriptional regulation, biosynthetic cofactor pathways, and bacterial community coordination, creating a resistance barrier requiring coordinated mutations across multiple unrelated systems. Furthermore, SME’s ability to disrupt biofilm formation addresses a critical limitation of current antibiotics, as biofilms can increase antibiotic tolerance, while SME’s dual action of directly killing planktonic bacteria and preventing biofilm establishment suggests potential efficacy against both acute and chronic infections.

Our study reveals SME’s antimicrobial potential, but highlights key limitations that require further investigation to fully validate its efficacy. The unavailability of Squama Manitis since June 2020 has limited SEM analysis on additional species (*A. hydrophila*, *A. lwoffii*, *P. aeruginosa*, and *B. cereus*) and testing against drug-resistant clinical isolates. Moreover, for these additional strains, we did not perform full MIC or MBC titrations; instead, we conducted fixed-dose growth inhibition assays. These were designed as preliminary screens to explore SME’s broader activity spectrum. Future work using the synthetic MAC4 formulation will include complete titration and statistical comparisons to assess interspecies differences in susceptibility. To overcome material and conservation constraints, we have developed a synthetic MAC4 formulation, which builds on SME’s mechanistic insights. Our *iScience* study identified 1,150 differentially expressed genes in *E. coli* treated with MAC4, with 372 overlapping with SME (e.g., *sdhA*, *sdhB*, *sdhD*, *sucA*, *pepN*, *rpoE*), reinforcing the consistency of our transcriptomic findings (Table S10) [43]. Future MAC4 studies will include complete titrations, statistical comparisons, and functional validation assays. Future research should also include time-course multi-omics studies to characterize dynamic effects and distinguish direct SME impacts from secondary stress responses, structure-activity optimization of the MAC4 formulation through synergy testing with conventional antibiotics, and essential in vivo efficacy studies to assess therapeutic potential, pharmacokinetics, and safety profiles [44]. Laboratory evolution and microbiome impact studies are essential to explore resistance and ecological outcomes. MAC4 enables validation of SME’s multi-target mechanism across diverse pathogens while supporting sustainable antimicrobial development without reliance on endangered species.

This work demonstrates the value of integrating traditional knowledge with modern systems biology approaches, where transcriptomic analysis not only validated traditional uses of SM but also revealed mechanistic insights that guide rational drug design applicable to other traditional medicines [12]. The conservation implications extend beyond pangolin protection to establishing a sustainable framework for natural product drug discovery that focuses on understanding and replicating bioactive mechanisms rather than harvesting endangered species, thereby preserving biodiversity while advancing medical science [45].

## 5. Conclusions

This study establishes that SME exerts broad-spectrum antibacterial activity through a unique multi-target mechanism, simultaneously disrupting bacterial metabolism, DNA repair responses, and cell envelope integrity. The identification of key metabolic pathways suppressed by SME—particularly the TCA cycle, fatty acid degradation, and pyruvate metabolism—provides a mechanistic foundation for its potent bactericidal effects against both Gram-positive and Gram-negative pathogens. In parallel, transcriptomic evidence reveals downregulation of the SOS response, DNA recombination, and damage-induced transcriptional programs, indicating that SME compromises bacterial genome maintenance and stress adaptation under hostile conditions. Critically, SME’s ability to penetrate outer membranes, suppress biofilm formation, and create synthetic lethality through coordinated pathway disruption addresses major clinical challenges in antimicrobial resistance. While validating traditional medicinal uses of SM, this work demonstrates how modern multi-omics approaches can decode ancient remedies to guide sustainable antimicrobial development without relying on endangered species. The convergence of transcriptomic, phenotypic, and metabolomic evidence presented here, combined with our synthetic MAC4 formulation, advances understanding of complex natural product mechanisms, while establishing a conservation-conscious template for next-generation bioinspired drug design that could help address the global antimicrobial resistance crisis.

## Figures and Tables

**Table 1:** MIC and minimum bactericidal concentration (MBC) values of SME against *E. coli* and *S. aureus*.

Bacteria Strain	SME	
	MIC	MBC
*E. coli*	31.25 mg/mL	31.25 mg/mL
*S. aureus*	31.25 mg/mL	62.5 mg/mL

## Data Availability

The data that support the findings of this study have been deposited into the CNGB Sequence Archive (CNSA) [46] of China National GeneBank DataBase (CNGBdb) [47] with accession number CNP0004243 and the BioProject PRJNA956429 for National Center for Biotechnology Information (nih.gov).

## Supplementary Materials

The following supporting information can be downloaded at: https://www.mdpi.com/article/10.3390/biology14080949/s1, Figure S1. SME’s antibacterial spectrum. Growth inhibition (%) of *A. hydrophila*, *A. lwoffii*, *P. aeruginosa*, and *B. cereus* at 20–40 mg/mL SME versus controls (mean ± SD; n = 3). Figure S2. SME’s cytotoxicity profile. Viability of human HaCaT cells after 24 h SME treatment (3.91–125.00 mg/mL) by CCK-8 assay (mean ± SD; n = 3). Table S1. Summary statistics of RNA-Seq data. Table S2. Summary statistics of filtered RNA-Seq data. Table S3. Summary statistics of clean reads comparison to reference genome. Table S4: List of upregulated gene in SME treatment group. Table S5: List of downregulated gene in SME treatment group. Table S6: List of GO term enriched in upregulated differentially expressed genes. Table S7: List of GO term enriched in downregulated differentially expressed genes. Table S8: List of KEGG term enriched in upregulated differentially expressed genes. Table S9: List of KEGG term enriched in downregulated differentially expressed genes. Table S10: List of 372 overlapping differentially expressed genes between SME and MAC4 treatments in *E. coli*.
