# Correction: Chen et al. Squama Manitis Extract Exhibits Broad-Spectrum Antibacterial Activity Through Energy and DNA Disruption Mechanisms. *Biology* 2025, *14*, 949

**DOI:** 10.3390/biology15151245

**Published:** 2026-07-28

**Authors:** Li Chen, Kunping Song, Mengwei Cheng, Aloysius Wong, Xuechen Tian, Yixin Yang, Mia Yang Ang, Geok Yuan Annie Tan, Siew Woh Choo

**Affiliations:** 1 Department of Biology, College of Science and Technology, Wenzhou-Kean University, 88 Daxue Road, Ouhai, Wenzhou 325060, China; s2040336@siswa.um.edu.my (L.C.); 1190912@wku.edu.cn (M.C.); aloysiusw@wku.edu.cn (A.W.); tianxuechen@wku.edu.cn (X.T.); yyang@kean.edu (Y.Y.); 2 Institute of Biological Sciences, Faculty of Science, Universiti Malaya, Kuala Lumpur 50603, Malaysia; 3 Zhejiang Bioinformatics International Science and Technology Cooperation Center, 88 Daxue Road, Ouhai, Wenzhou 325060, China; 4 Zhejiang Province-Malaysia International Joint Laboratory for Modern Agriculture and Microbial Innovation, Wenzhou-Kean University, Ouhai, Wenzhou 325060, China; 5 Department of Diagnostic and Allied Health Science, Faculty of Health and Life Sciences, Management and Science University, Shah Alam 40100, Malaysia; angmiayang@msu.edu.my

In the original publication [1], there was an omission in the Materials and Methods Section (Subsection 2.1, Sample Preparation). The original text did not include an explicit ethical and conservation context statement regarding the use of pangolin scales.

A correction has been made to Section 2. Materials and Methods, Subsection 2.1. Sample Preparation, Paragraph 1:

“All Squama Manitis (SM) samples were purchased before June 2020 from Tong Ren Tang pharmaceutical company (Beijing, China). These samples were obtained from legally marketed traditional medicine products purchased through commercial channels in China. No wild pangolins were collected, handled, or harmed for the purpose of this research, and no additional trade in pangolin-derived materials was conducted as part of the study. This work does not promote the medicinal use of pangolin scales. Instead, it aims to characterize their bioactive components and antibacterial mechanisms to support the development of sustainable alternatives that may help reduce demand for wild pangolin products and contribute to species conservation. Sample preprocessing followed established protocols with modifications [16]. Briefly, SM samples were surface-sterilized by vigorous rinsing and wiping with 75% ethanol, followed by sterile water washing to remove surface contaminants. Samples were dried in a 60 °C incubator overnight, then pulverized using a sterile masher (Little Bear Electric Co., Foshan, China) that was pre-sterilized with 75% ethanol. The resulting powder was collected under sterile conditions and stored at −20 °C until use in subsequent experiments.”

The authors state that the scientific conclusions are unaffected. This correction was approved by the Academic Editor. The original publication has also been updated.
