# Supplementary material for: Squama Manitis Extract Exhibits Broad-Spectrum Antibacterial Activity Through Energy and DNA Disruption Mechanisms
Source: Biology (Basel). 2025 Jul 28;14(8):949. doi: 10.3390/biology14080949 (PMC12383583; doi:10.3390/biology14080949)
Supplement: Supplementary file 1 [file biology-14-00949-s001.zip › Supplementary figures.pdf]

Supplementary figure:

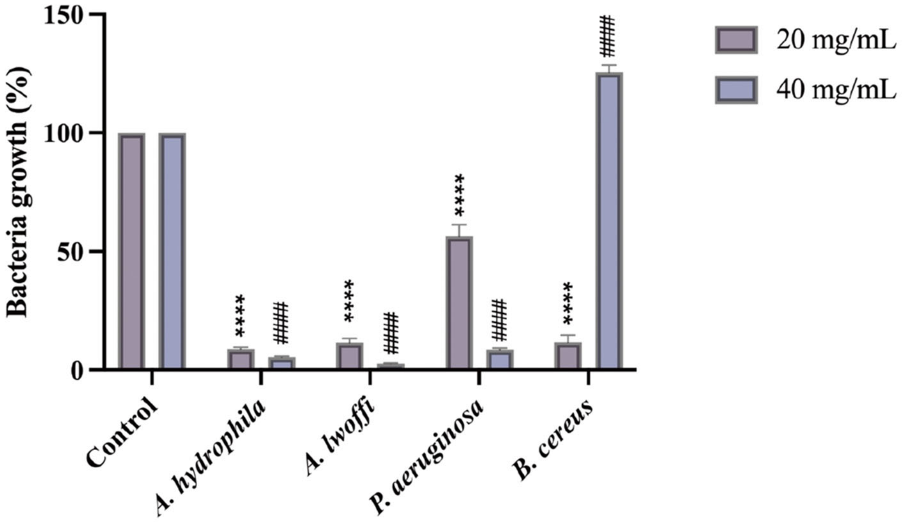

Figure S1. SME's antibacterial spectrum. Growth inhibition (%) of *A. hydrophila*, *A. lwoffii*, *P. aeruginosa*, and *B. cereus* at 20-40 mg/mL SME versus controls (mean ± SD; n=3).

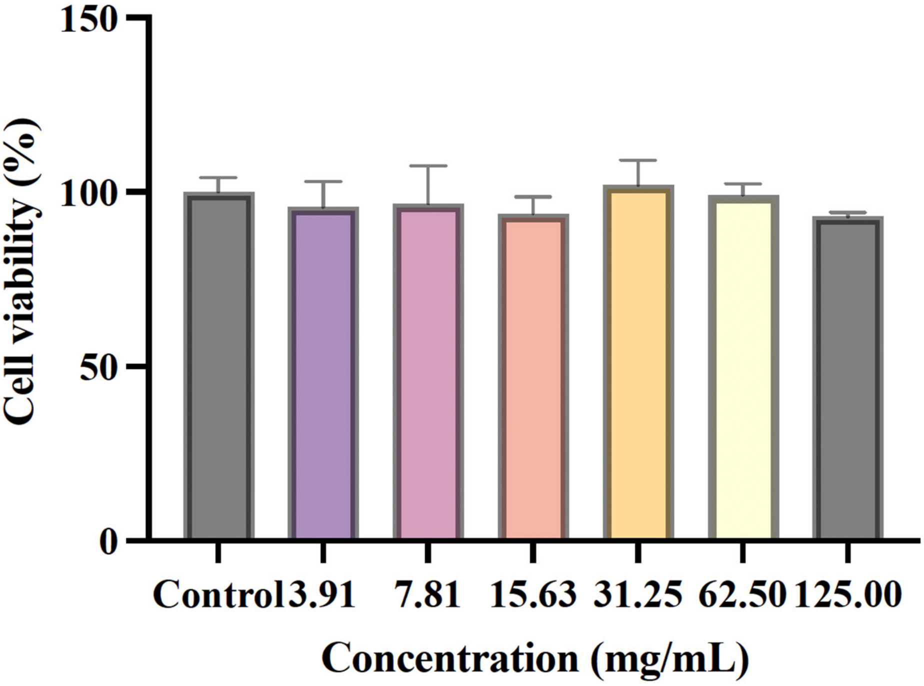

Figure S2. SME's cytotoxicity profile. Viability of human HaCaT cells after 24 h SME treatment (3.91-125.00 mg/mL) by CCK-8 assay (mean ± SD; n=3).
